# The Art of Waiting Humbly: Women Judges Reflect on Vertical Gender Segregation

**DOI:** 10.1007/s10691-023-09533-w

**Published:** 2023-08-18

**Authors:** Marína Urbániková, Barbara Havelková, David Kosař

**Affiliations:** 1 Judicial Studies Institute, Masaryk University, Brno, Czech Republic; 2 Faculty of Law, University of Oxford, Oxford, UK

**Keywords:** Central and Eastern Europe, Czech Republic, Gender, Judiciary, Vertical gender segregation, Women judges

## Abstract

Central and Eastern European countries (CEE), compared to common law countries but also other civil law countries of Europe, are known for a strikingly high representation of women within judiciaries. This, however, does not mean that equality has been achieved, as women judges do not reach leadership positions at the same rate as their male peers. Taking the Czech Republic as a case study, this contribution explores the barriers women judges face within a CEE judiciary and analyses their reflections on their positions. The interviews with women judges show that while they are well aware of what is holding them back, most of them do not perceive the structurally unequal position of men and women in Czech society and in the judiciary as a problem and accept the consequences as being part of women’s destiny. This means that the system currently lacks bottom-up incentives and pressure for change.

In December 2021, the President of the Czech Supreme Administrative Court (SAC) reached the compulsory retirement age and Czechia searched for a new head of the administrative judiciary. Barbara Pořízková, SAC’s female Vice-President, was a primary contender. However, the Czech President eventually chose a male President of SAC in January 2022. Yet again, one could say. The same story took place in 2019 and 2020, when male candidates managed to succeed in the contest for a Supreme Court President as well as for two presidents of high courts. Women judges are thus missing in the key leadership positions within the Czech judiciary. But women judges often do not make it to apex courts in the first place. Even though women constitute almost two-thirds (61%) of the Czech judiciary, they are clearly underrepresented in the positions of power, both in the higher courts within the court hierarchy and in managerial posts within the court administration: for example, they constitute only 20% of Supreme Court judges and 43% of the court presidents (Havelková et al. 2022).

Beyond the intrinsic value of having more women in key decision-making roles, gender diversity on the bench is important as it may improve legitimacy and public confidence in the judiciary and provide mentoring by women judges for other women in the legal profession (Clayton et al. 2019; Grossman 2012; Malleson 2003; Rackley 2013). More practically, women judges are also likely to have more empathy with women litigants and witnesses, educate and civilise their male colleagues on gender issues, and bring a gendered sensibility to the process of decision-making (Hunter 2015). This is especially important in post-communist countries like Czechia, where people’s trust in the judiciary was low (Blisa et al. 2018) and gender stereotypes widespread (Havelková 2017) after the collapse of communism.

Nonetheless, Czech women judges are clearly prevented from proceeding as smoothly as their male counterparts within the judiciary. This is not a novel problem. It permeates many judiciaries in Europe as well as worldwide. There is, in fact, a burgeoning literature analysing the lack of career progress of women in the judiciary, often based on interviews with female judges as a major method to identify the career obstacles (e.g., Bessière and Mille 2014; Boigeol 1993; Durant 2004; Leith and Morison 2013; Levinson and Young 2010; Schultz 2003, 2013; Schultz and Shaw 2008, 2013a; Schultz and Masengu 2020; Valdini and Shortell 2014).

However, most of the literature focuses primarily on the consolidated democracies, and Western Europe in particular, with recent additions covering Asia–Pacific (Crouch 2021), Africa (Jarpa Dawuni 2021) and Australia (McLoughlin 2021). Only a few articles deal with the different ceilings for women judges in the developing democracies (Achmad and Halimatusa’diyah 2022; Bonelli 2015; Halilović and Huhtanen 2014; Jarpa Dawuni 2021; Kalem 2020; Masengu 2020) or authoritarian states (Cardinal 2008; Uzebu-Imarhiagb 2020; Zheng et al. 2017). In-depth studies on the career progress of women judges in post-socialist countries in Central and Eastern Europe (CEE) are virtually non-existent (one exception being Halilović and Huhtanen 2014). And only a few papers have discussed female judges as part of a wider discussion of women in legal professions (e.g., Fuszara 2003; Shaw 2003; see also Havelková et al. 2022 and references therein). Yet it is important to understand what barriers and obstacles women judges face within the CEE judiciaries, since their problems differ in many respects from those of Western Europe. Compared to common law countries, but also other civil law countries of Europe, their representation within judiciaries is strikingly high: it is 69% in post-socialist countries of Europe compared to the continent’s 61% and the EU’s overall 53% (CEPEJ 2018). This, however, does not mean that equality has been achieved as they clearly do not reach the leadership positions at the same rate as their male peers (see, e.g., CEPEJ 2020). Our article aims to fill this gap by providing the first interview-based study of vertical segregation in the judiciary in post-socialist CEE that also analyses the lack of reflection of one’s own position by women judges.

We show that while the research participants are very well aware of what is holding women judges back, a majority of them take the status quo for granted, do not perceive the structurally unequal position of men and women in Czech society and in the judiciary as a problem, and accept the consequences of this inequality as a part of a woman’s fate. We argue that this rationalisation of the under-representation of women judges in the top echelons of the Czech judiciary, the lack of reflection on its deeper reasons, and, in some cases, even the denial of the existence of any gender inequality not only contribute to the status quo but help reproduce it.

While our results cannot be easily generalised to other CEE countries due to some differences in legal culture and factual background, we provide an analytic toolbox that can be applied to other countries in the region. Our toolbox also challenges studies concerning Western Europe. While some scholars there identified a genuine lack of interest in reaching a top position (Boigeol 1993) and lack of self-confidence (Schultz 2013) as the reasons behind slower career advancement of women judges, women judges’ (critical) reflections on their situation have been underexplored (for an exception relating to another legal profession, see Hunter 2002 and her study of women barristers’ denials of discrimination at the Bar). Do women judges interpret the fact that they are not reaching the top echelons as unfair and unjust, and, at large, as an issue to be addressed? To eliminate vertical segregation in the judiciary, it must first be perceived as a problem. The perception of gender issues and gender relations should thus be considered as a potentially important factor also in jurisdictions well beyond CEE.

## Context and Literature Review

In this section, we situate our research in the existing literature. First, we present findings from existing socio-legal literature on gender and career progression in the judiciary. Subsequently, to give the reader sufficient context, we present background information on feminism and the situation of women in the CEE region and in Czech society, and on the system of the Czech judiciary.

### Gender and the Judicial Career

Analysis of the limited access to and progress of women in the world’s judiciaries have grown in number and country coverage over the past two decades (e.g., Schultz and Shaw 2003; Schultz and Shaw 2013a, b; Schultz and Masengu 2020). The issue of access has been the focus mainly of common law scholars (Barmes and Malleson 2011), as the proportion of women, at around 30%,^1^ is roughly half of that found in civil law countries of continental Europe (Schultz 2013). Here, recruitment out of the legal profession, as opposed to a system of a career judiciary, creates specific barriers. For example, a limited understanding of merit as consisting mainly of qualities possessed by the Bar has excluded women at a higher rate (Leith and Morrison 2013).

In most civil law countries, women constitute the majority of judges—the average in civil-law countries of the EU is 61% (CEPEJ 2018). The focus has, thus, been on barriers to career progress (Boigeol 2013; Schultz 2003, 2013) as women encounter both horizontal (Schultz 2003) and vertical segregation (Schultz and Masengu 2020 and references therein; for Czechia see Havelková et al. 2022). The question of promotion is of growing interest in the common law world too (Durant 2004), with scholars increasingly noting the “leaky pipeline” (e.g., for the USA, Gertner 2012) or “glass ceiling” (e.g., for UK, Guyard-Nedelec 2018) for women within the judiciary.

Previous studies have identified a number of barriers to women’s access to and progress in the judiciary.^2^ First, selection and appointment processes may indirectly disadvantage women. In particular, lack of transparency appears to have a negative effect on women’s representation in leadership positions. A cross-jurisdictional study of high courts by Valdini and Shortell (2014) suggests that women are more likely to be appointed via “exposed” selection processes rather than “sheltered” ones. A lack of transparency seems to plague common law countries more (IDLO 2018) than civil law ones (Schultz and Shaw 2008).

Second, previous research suggests that selection and promotion processes are sometimes marked by gender discrimination, sexism, stereotypical thinking, and gender bias on the part of gatekeepers (Achmad and Halimatusa’diyah 2022; Chan 2014; Durant 2004; Feenan 2008; Kalem 2020; Levinson and Young 2010; Roche 2003). This can take the form of the undervaluing of women’s qualifications (Schultz and Shaw 2013b), seeing the judicial profession as masculine (Kalem 2020; Levinson and Young 2010), and perceiving women as unsuitable, or at least less suitable and qualified, for judicial office (Chan 2014; Roche 2003). But a lack of sisterhood has also been noted (Schultz 2013).

Third, even if the selection and appointment processes are not directly marked by discrimination and prejudice, they often rely heavily on informal networks to which women have less access (Virtue Foundation cited in IDLO 2018). Men have more opportunities for informal socialising and interactions and more easily form “old boys’ clubs” where they acquire social capital that gives them career advantage (Chan 2014; Kalem 2020; Shen 2020). These gendered informal institutions, such as *guanxi* (social exchange) in China (Zheng et al. 2017) and gentlemen’s pacts in Mexico (Pozas-Loyo and Rios-Figueroa 2018), can take country-specific forms.

Fourth, another problem is the structurally disadvantaged position of women in the judiciary and legal professions. In case of appointments and promotions, the size of the pool of eligible applicants is usually larger for men than for women, and there are usually fewer women lawyers than men with legal background and credentials that are considered sufficient (Barmes and Malleson 2011; Martin 2004; Masengu 2020; Uzebu-Imarhiagb 2020). It is not only the social construction of merit that comes into play here (Leith and Morrison 2013), but also horizontal gender segregation in the legal field and in the judiciary. Women often hold less prestigious positions and focus on areas of law (such as family or employment law) that have less prestige and fewer career opportunities (Havelková et al. 2022; Roche 2003; Treanor 2020).

Finally, while women as a group appear to be disadvantaged by the previously listed barriers and mechanisms, mothers and primary carers (who are overwhelmingly women) face further challenges concerning work-life balance (Boigeol 2013; Bonelli 2015; Chan 2014; Durant 2004; Duarte et al. 2014; Halilović and Huhtanen 2014; IDLO 2018; Roche 2003; Schultz 2003; Schultz 2013; and references therein; Achmad and Halimatusa’diyah 2022; Kalem 2020; Shen 2020). These studies from different countries and regions show that traditional gender norms that consider men as breadwinners and women as homemakers still prevail, and that women are still expected to prioritise motherhood and caring responsibilities over careers. Therefore, it is women who interrupt their careers to take care of children more often and for longer periods of time and who carry out most of the unpaid work in the home. Logically, they do not have the same amount of time available to develop their careers as their male colleagues. Moreover, they also tend to adjust their career expectations and ambitions to this gendered division of paid and unpaid work (Roche 2003; Treanor 2020).

Indeed, a previous paper on the Czech judiciary also noted that the gendered division of labour, especially in the home, needs to be viewed as a central reason for vertical segregation in the Czech judiciary, with women experiencing a considerable motherhood penalty due to both the long absences during maternity and parental leave as well as the additional responsibilities if they become the primary child caregiver thereafter (Havelková et al. 2022).

There are some additional extra-judiciary reasons why promotion, especially to a higher court, might be more difficult for primary carers. A notable one is the fact that the move to a higher court is often also a geographical move for which women are less likely to have support (Schultz 2003; see also Virtue Foundation cited in IDLO 2018), partly due to the lack of support in “dual career couples” (Schultz 2013: 160). But concrete obstacles also exist within the judiciary, for instance, a lack of accommodation for primary carers, such as insufficient availability of part-time positions and their incompatibility with promotion (Schultz 2013). Stereotypical assumptions about women’s lack of interest in managerial duties, secondment or promotion to a higher court once they have children seems to be a more general problem (Schultz 2013).

A question which has so far been relatively marginal in these studies relates to women judges’ (critical) reflections on their situation and on (the constraints on) their choices and preferences. Ulrike Schultz (2013, 161–163) has identified “inner career obstacles and career renunciation” as one of the impediments to career progress for women. Her respondents observed that women lack self-confidence and need to be encouraged (Schultz 2013). The question arises to what extent do women perceive this as a gender-based disadvantage. In other words, to what extent is this seen as an individual problem for the women to overcome or a structural problem to be addressed at the level of the institution. The relatively underexplored question of women judges’ reflections is a crucial one in our paper.

### Women and Gender in the CEE Region and in Czechia

A question one might ask is whether CEE judiciaries evince any unique traits in relation to gender. Notably, the overall average representation of women in the judiciary is higher in post-socialist countries of Europe: 69% compared to the continent’s 61% and the EU’s overall 53% (CEPEJ 2018). The explanation is at least two-fold. First, women made the majority of judiciaries in many state-socialist countries when the job was technical, poorly paid, and non-prestigious (Fuszara 2003; Havelková et al. 2022). Second, immediately after the fall of the regime in 1989, many male judges left for better-paid positions in the private sector, while the sudden need for more judges was filled with women, sometimes from positions of in-house lawyers who were made redundant due to changes in the economy (Havelková et al. 2022).

While this explains the presence of women, it does not explain their relative absence in positions of power in the judiciary. This is not specific only to the Czech judiciary or the Czech labour market in general, and it has deeper causes stemming from the historical socio-cultural context common to the CEE region. Thus, a brief introduction of these follows.

Despite perceptions that state-socialist countries, including Czechoslovakia, were beacons of women’s emancipation and equality, the reality was different in three important ways. First, there was “public equality”—especially in education and employment—but “private difference” as gender relations in the home remained untouched. Under state socialism, the issue of gender equality and emancipation was framed primarily as a question of the equal role of women and men in economic production, not as a question of individual rights (Pascall and Kwak 2010). While the socialisation of care (e.g., extensive network of nurseries and kindergartens) helped women to join the labour market, domestic work remained their responsibility, leading to a double burden of paid and unpaid work for women which persists to this day in the CEE region (Ukhova 2020).

Second, and relatedly, the traditional symbolic gender order remained unchallenged because the state socialist countries institutionalised it based on patriarchal principles (Jezerska 2003; Metcalfe and Afanassieva 2005). As gender equality was constitutionally enshrined, the states enforced a gender-neutral policy and almost an asexual approach to gender relations while “gender inequalities were largely silenced and repressed within public discourse” (Metcalfe and Afanassieva 2005, 400). So, if any progress was made, it was limited to the material sphere; a situation which has largely not changed until today.

Third, women had limited resources to challenge male power because they were unable to organise in civil society without state oversight (Metcalfe and Afanassieva 2005). Also, due to the rather comprehensive intellectual isolation, they did not have an opportunity to engage with international feminism of the second wave which brought insights into the social-constructivist nature of gender and the systematic nature of patriarchy (Havelková 2017).

Thus, the post-1989 economic and political transition and transformation began with the inherited laws and policies that were actually gender-conservative (pro-motherhood and pro-family) rather than gender equalising (Havelková 2017). The transition to a market-based systems brought about erosion of state childcare services which in the past enabled women to participate in the labour market; this, too, contributed to a heavy “motherhood penalty” in the CEE countries (e.g., Cukrowska-Torzewska Matysiak 2020). Beverly Dawn Metcalfe and Marianne Afanassieva (2005, 397) even argue that the post-1989 changes gave rise to a process of remasculinisation which reaffirmed “gendered hierarchies and gender power relations in public and private realms”.

Under such conditions and in such an environment, feminist ideas are only slowly gaining ground. Gender insensitivity is traditionally rooted in most countries of the region (Jezerska 2003), feminism as a movement and as an ideology remains resented and stigmatised (Forest 2006), and considered as being against free choice and market competition (Metcalfe and Afanassieva 2005).

Thus, if we now turn specifically to the Czech context, in material terms, the situation of women and various gender gaps which have been inherited from state socialism persisted, and sometimes even worsened after 1989 (Havelková 2017). Until today, Czech men and women differ significantly in terms of time spent in paid and unpaid work (European Commission 2017), and women are overwhelmingly the primary caregivers in the Czech Republic (for instance, they constituted 98% of the parental benefit recipients in 2020; Czech Statistical Office 2021a). This, together with the deep-seated social norm that a good mother should stay at home taking care of her child for at least 3 years, leads to a long-term drop-out from the labour market (Bičáková and Kalíšková 2015). The gendered division of paid and unpaid labour feeds into an unequal representation of men and women in positions of power (Czech Statistical Office 2021b) and a considerable gender pay gap (Eurostat 2021).

In terms of the symbolic order, Czechia inherited an uncritical, unreconstructed, largely essentialist understanding of differences between men and women (Havelková 2017), and this has shifted little over time. To this day, Czech society still holds rather traditional beliefs regarding male and female roles, according to which women are predominantly (natural) caregivers and men (natural) breadwinners (CVVM 2020). Second, Czechia has a serious “‘no problem’ problem” (Rhode 1997) when it comes to gender inequality: the fact that a gender disparity exists, that it is an injustice, and one which might require a systemic solution is not very much realised, especially among the legal community (Havelková 2017).

### Women in Czech Courts

**Fig. 1:**
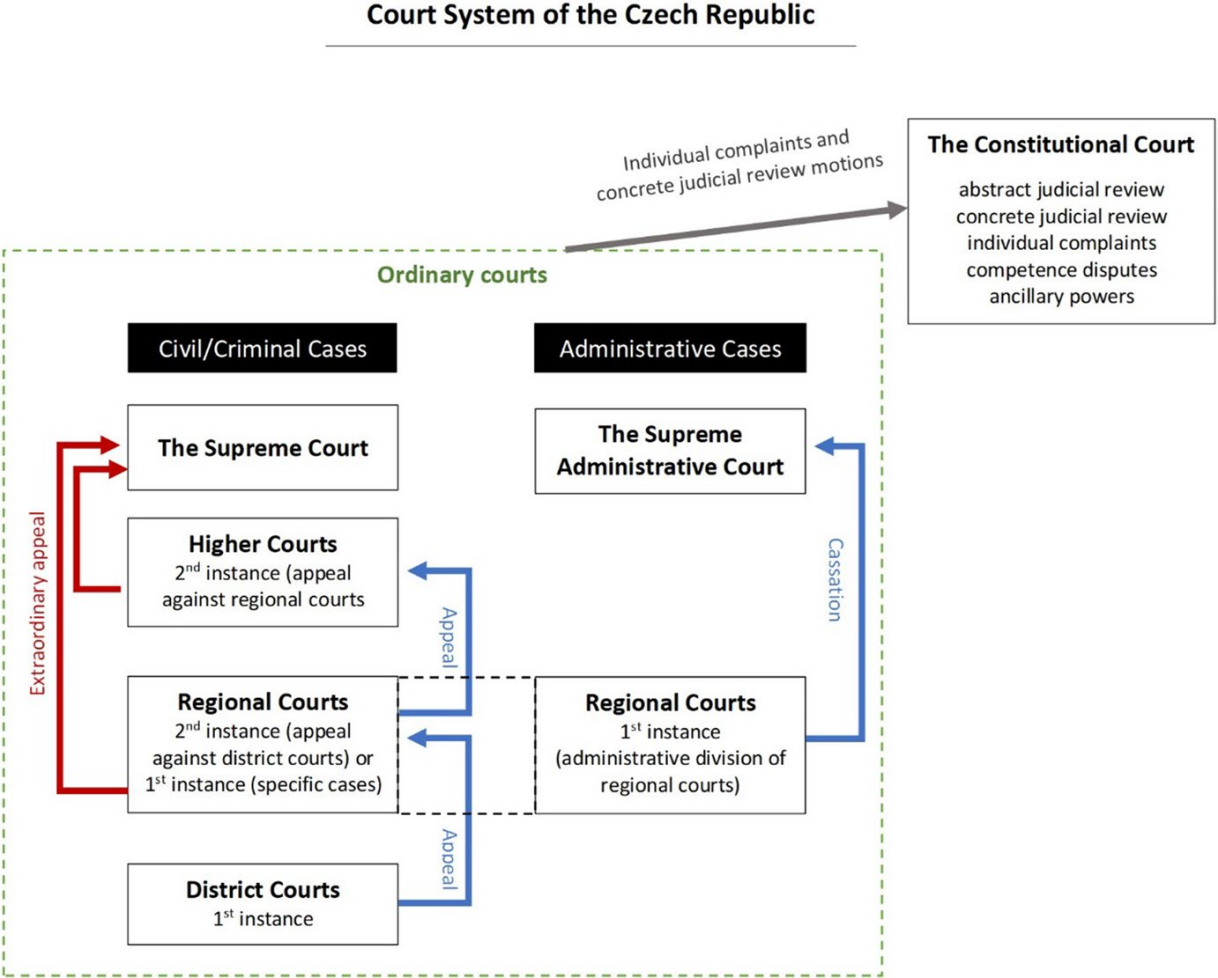
Czech court system. *Source*: authors

The gender stereotypes and the gendered division of labour also permeate the Czech judiciary and translate to the division of tasks between female and male judges. The higher we go in the court hierarchy the fewer women we see. First, in 2019, women made up 67% of district courts judges, 54% of regional courts judges, 44% of high courts judges, 31% of Supreme Administrative Court judges, and 20% of Supreme Court Judges (Havelková et al. 2022). Second, the same pattern is also visible when it comes to managerial positions in the court administration. In 2019, female judges made up 61% of regular (ordinary) judges, 47% of the court vice-presidents, and 43% of the court presidents (Havelková et al. 2022). What is more, women primarily hold the less interesting and the less visible posts of district court presidents, whereas men hold the key, powerful posts at the regional and apex courts (Havelková et al. 2022). Hence, although the first condition of gender equality—a fair representation of female judges in the judiciary as such—has been fulfilled in the Czech Republic, the Czech judiciary can at the same time work as a textbook example of vertical gender segregation. To contextualise these numbers within the hierarchy of the Czech judicial system, the court system in the country is presented in Fig. 1.

Given that women are clearly underrepresented at the highest levels of the Czech judiciary, it is important to take a closer look at its promotion rules and policy. Since Czechia inherited a bureaucratic career model of the judiciary (Bobek 2015), promotion of judges is a crucial tool that may have significant gender consequences for the careers of individual judges as well as for the composition of the judiciary at each of its tiers.

The first feature of the Czech career model of the judiciary is that, in contrast to common law countries that follow the recognition model (Garoupa and Ginsburg 2011), Czech judges enter the judicial ranks soon after graduating from the law school, usually at the lowest tier of the judiciary, and then try to climb to the upper echelons of the judicial hierarchy (Kosař 2016). Direct lateral appointment to higher courts from other legal professions is very rare (Bobek 2015). In such an environment it is promotion, not initial appointment, which determines the gender composition of the Czech judiciary from the level of appellate courts up. In other words, even if female candidates have the same fair chance to become judges (of the first instance court) as their male competitors, it does not mean that female judges, once appointed, have the same chance to get to the appellate or apex court.

In sum, the rules concerning promotion of judges are rather vague and leave significant decision-making leeway for certain key actors (Kosař 2016). For promotion to a regional court or a high court, the Law on Court and Judges stipulates at least 8 years of practicing law.^3^ Promotion to the Supreme Court^4^ and the Supreme Administrative Court^5^ requires at least 10 years of practice. The only substantive criterion is that a promoted judge must have sufficient legal skills and experience to ensure the proper exercise of the judicial office at a higher court to which she is promoted.^6^

On paper, the key role in promoting judges to higher courts is vested with the Minister of Justice, who needs to consult the relevant court presidents (of the court to which as well as the court from which a judge is being promoted). Different rules apply for promotion to the Supreme Court and the Supreme Administrative Court as the consent of the President of a given apex court is required.^7^ This rule results from the absence of a judicial council and serves as a bulwark against potential executive interference with apex courts. However, in practice it is the court presidents who de facto select judges to be promoted (Blisa et al. 2018; Kosař 2016) and the Minister of Justice merely rubberstamps their decision (Kosař 2017).

Usually, there are no open calls to fill the vacancies at higher courts which would allow lower court judges to lodge their promotion applications for the advertised position. The whole process of selecting the suitable candidates is rather informal and often preceded by temporary assignment to a given court prior to indefinite promotion (Kosař 2016). This temporary assignment to a higher court—“secondment”—serves as a testing period during which a court president and judges of a higher court can watch the candidate for promotion and make their assessment (Bobek 2015). During the secondment, the seconded judge temporarily becomes a judge of a higher court, is assigned case files as a judge rapporteur, and sits on panels with other judges with full voting rights. The rules of temporary assignment to appellate and apex courts vary from one court to another. Apart from the Supreme Administrative Court (Supreme Administrative Court 2014), there is no formal procedure on how to apply for temporary assignment. Instead, it is decided on an ad hoc basis, usually by a “tap on a shoulder” of a lower court judge by a president of a higher court where vacancies occur.

The second feature of the Czech career model of the judiciary is a strong role of court presidents: they have a major say in the selection, promotion and disciplining of judges as well as in other dimensions of judicial governance (Kosař 2016). Therefore, it is important to understand how court presidents and vice-president are selected. From 2011 onwards,^8^ there has been an open competition for filling each post of the court president and the vice-president (Derka 2017; Kolumber 2021). These rules were amended in 2014, and for each vacancy there is an ad hoc selection committee that consists of five members (three court presidents or vice-presidents and two members of the Ministry of Justice). A competition is announced in advance and the selection committee follows fair rules, including assessment of the clear criteria set in advance, standardised CVs, and random choice of the order of interviews.^9^

Different rules apply for the two apex courts, the Supreme Court and the Supreme Administrative Court. The President of the Czech Republic chooses a president and a vice-president from the judges of each court respectively. The Czech President has absolute discretion; there is no selection commission, and no formal procedure which candidates can use to apply. The selection happens behind the scenes after informal talks between the judicial leadership and the presidential aides. This practice has not changed since 1993, despite heavy criticism of the process of selection of the Supreme Court president in 2020 and the Supreme Administrative Court president in 2022 by the media and scholars (Nahodil 2020; Svobodová and Procházková 2022; Vaculík 2020).

## Method and Data

To learn more about the mechanism of vertical gender segregation in the Czech judiciary, we explored the perspectives and experiences of those who have first-hand experience of its negative effects: female judges. The study asks two research questions: 1. What, from the perspective of Czech female judges, are the main barriers and obstacles in the advancement of women to the highest positions in the judiciary? 2. How do they reflect on and interpret the roots and sources of these barriers and obstacles? To answer these questions, we used the method of semi-structured interview, “a qualitative data collection strategy in which the researcher asks informants a series of predetermined but open-ended questions” (Ayres 2008, 810).

We intended to have the maximum variety in the sample. We interviewed 14 judges from different regions of the Czech Republic and various types of courts. Four of the research participants hold or held managerial positions (court president or vice-president), and the rest are ordinary judges. The participants’ ages ranged from 37 to over 70 years (three of them have already retired). The minimum age for appointment as a judge is 30 (but in practice, newly appointed judges are often older); since the study examines vertical segregation, we addressed judges who already had enough experience to consider seeking promotion. The majority (11 out of 14) have children. None of the judges we asked for an interview declined to participate in the research.

A written interview guide with a list of topics to be covered was developed in advance. Once informed consent was obtained from the participants, interviews were conducted by one of the authors in a variety of locations (mostly in the participants’ offices, but also in nearby cafés or at the participants’ homes; two interviews were conducted online) from July 2018 to December 2019.^10^ The interviews took 90 min on average.

The interviews were recorded, transcribed verbatim, and analysed using the ATLAS.ti software kit. Thematic analysis, “a method for identifying, analysing and reporting patterns (themes) within data” (Braun and Clarke 2006, 79), was used to organise and describe the dataset. The authors coded the interviews and consolidated codes into several content domains using the inductive approach. To ensure privacy and anonymity, due to the very small number of female judges in the top echelons of the Czech judiciary, only limited information on research participants is provided throughout the text: their age groups (younger generation, i.e., up to 45 years; middle-aged generation, i.e., between 46 and 60 years; higher-age generation, i.e., over 60 years) and the court level (district/regional/municipal court—eight judges; apex court, i.e., the Supreme Administrative Court, the Supreme Court, the Constitutional Court—six judges; in the case of retired judges, their most prestigious position was taken into account).

## Data Analysis and Interpretation: Czech Female Judges and Their Thorny Path to the Top

In the following text, based on the interviews with 14 Czech female judges of different ages, in different positions, and from various courts, we describe what they see as the main obstacles to career advancement and how they interpret the roots of those barriers. While the first part summarises the views and perspectives of the research participants, the second part offers a critical interpretation and wider contextualisation of how Czech female judges reflect on vertical gender segregation.

### Perceived Barriers and Obstacles to Career Advancement: Family, Lack of Ambitions, and Nontransparent Promotion Policy

The research participants identified five key factors that hinder the career advancement of female judges. Ranked according to the frequency of mention, they are: 1. family obligations, 2. lack of ambition, 3. nontransparent promotion policy and its consequences (importance of informal networks and ‘old boys’ clubs’; more room for implicit bias in promotion; difficulties in career planning), 4. restricted mobility, and 5. the lack of targeted support for women’s leadership. All the research participants agreed that female judges do not advance to more prominent positions in higher numbers because *they are the family caregivers and household managers*. Given the already described dominant gender norms resulting in an unequal division of paid and unpaid work in Czech society, this finding is hardly surprising. As the bulk of household chores, childcare, and care of other family members is borne by women, they have less time and energy to pursue their careers. This was acknowledged by literally all the interviewed judges:

[…] men of the same age, around the age of 35, 40, seem to be interested in career advancement. While the primary care of the children is still on a woman, she has to deal with kindergarten, school all the time, and when she comes in from work she must organise it all.

A district/regional/municipal court judge; younger generation

At the same time, the research participants mentioned that due to the domestic burden, women *lack ambition* and *are not so interested in career growth*. Virtually all the interviewed judges stated, in variants, that women are not ambitious enough, less ambitious than men, or just not ambitious at all:

[T]hey are so busy that then they no longer think about career advancement, or that later on, they don’t want to, they have their current job, they have their family, and then they don’t want to go anywhere higher.

A district/regional/municipal court judge; younger generation

The next three factors identified by the research participants are closely related to the specificities of the Czech judiciary. Promotion in the judiciary can take two forms: either advancement to a higher court or gaining a managerial position (court president or vice-president). Transfers to higher courts entail the need to move to another city where the higher court resides. The three top Czech courts—the Supreme Court, the Supreme Administrative Court, and the Constitutional Court—are located in Brno, the second biggest city in the Czech Republic, located roughly 200 kms from Prague, and judges based in other regions are often reluctant to relocate. As stated by the interviewed judges, moving to another city is more difficult for women because, according to the usual scenario, a woman (and the rest of the family) follows a man and not the other way round. Thus, *mobility* is a challenge in particular for female judges and this contributes to curbing their ambitions. The deep entrenchment of gender norms is evident also from the strong language used by some of the research participants who repeatedly described commuting or a change of residence as “impossible” for women:

But when there are small children, it is absolutely impossible for a woman to commute [to a higher court in another city], and even if those children are older. Imagine that if a woman has a family, all of a sudden she would have to leave everything and go there [to another city] for a week or part of a week. A man can do that.

An apex court judge; higher-age generation

Next, while there are open competitions for filling the posts of court presidents, the procedure for promoting judges to higher courts is unclear and does not have formal rules (see above). *The lack of a transparent promotion policy* in this respect means that judges, both men and women, may not know how to proceed when interested in career advancement. According to the research participants, it is usual to be seconded to a higher level court before transferring there for good. Again, the problem is that there are no formal rules for these secondments. Ambiguity was obvious from the responses of the district and regional courts’ judges when asked about their career aspirations. Even those who stated that they would have the ambition to transfer to a higher court admitted that in fact they do not know how to proceed:

Interviewee: […] but I would definitely like to try the insolvency agenda, at least in the form of a secondment. Most of these cases are dealt with by [a higher court name].

Interviewer: How does that work in [city name]? Can you formally apply?

Interviewee: You can ...

Interviewer: And to whom, to your court president or the president of the higher court?

Interviewee: Of the higher court, but ...

Interviewer: Is it discussed informally beforehand, or is it better to ask the court president beforehand?

Interviewee: I don’t know.

Interviewer: And is it even standard for an ordinary judge to meet with the president of a higher court?

Interviewee: I can’t answer that; I don’t know.

A district/regional/municipal court judge; younger generation

According to the interviewed judges, the lack of transparency in promotion to higher courts has three negative consequences that mostly impact women. First, it increases the importance of *informal personal networks* that women have less time to build and develop. These networks are of value in the case of all sorts of promotion, but the lack of formal promotion rules in the case of advancement to higher courts makes them particularly salient. Several research participants mentioned the significance of personal relationships in promotion to higher courts:

It is a question of that particular offer in that particular court, which is being dealt with by the president of that court. And it is true that even in the judiciary, it is important who is friends with whom, who is close to whom.

An apex court judge; higher-age generation

Second, the existence of *the old boys’ clubs* and the tendency of male decision-makers to choose other men for the top positions were identified by the research participants as another obstacle in the promotion of female judges to higher courts. As management positions are predominantly occupied by men, the lack of formal rules leads to a situation where *men tend to overlook women and select other men*, also due to *unconscious and implicit bias*. Again, it can be argued that this is a wider phenomenon when it comes to promotion, but the non-existence of formal promotion policy makes it more salient:

The system looks neutral, but the fact is that men prefer men for those high positions.

An apex court judge; middle-age generation

Because it’s just like that, it is closer to them [to select a man rather than a woman].

A district/regional/municipal court judge; middle-age generation

Logically, given the quantitative dominance of female judges in the Czech judiciary, the question arises why they do not form their own clubs and networks. The lack of leisure time is just one part of the explanation. Equally importantly, gender as a topic is only slowly and gradually opening up in the Czech judiciary, which is reflected, among other things, in the nonexistence of an association of female judges, or at least a women’s section of the Czech Union of Judges.

Third, the lack of formal rules for promotion to higher courts makes *career planning* more difficult. As the criteria for promotion can only be guessed at, the whole process is unpredictable and cannot be planned in advance, which is a challenge for the reconciliation of work and family life. As explained by one of the research participants, when a judge is approached with an offer, it is not possible to anticipate it or postpone it, and the offer will likely not be repeated:

As far as those levels of court are concerned, the lack of any promotion rules is really to blame [for vertical gender segregation]. […] when a woman is approached with an offer, she may be approached at a time when it [the promotion and relocation] doesn’t suit her, and no one will actually approach her a second time. If there were rules, they could say well, you can’t now, and what about in a year and a half? […] a woman needs to plan things in advance because of the family.

A district/regional/municipal court judge; middle-age generation

Several of the identified factors that hamper female judges’ career advancement are not unique to the judiciary, and as such they cannot be fully resolved within the judicial system. However, the current situation could be at least partially remedied if the decision-makers actively encouraged women to strive for promotions and apply for higher posts. Unlike in some other professions, there is *a lack of targeted support for women’s leadership* in the Czech judiciary. As pointed out by several research participants, the extra push that is often needed to motivate reluctant female adepts is simply missing:

The problem with this court is that no one is trying much to attract women here. […] Many women would like to be here, but it’s a question of politics, for example, who the court wants to be here and who it doesn’t want.

An apex court judge; middle-age generation

Or they [women] are little addressed with offers. […] The bosses should also look around a bit today and keep in mind that women with their life stories bring different perspectives to the justice system.

An apex court judge; higher-age generation

Several judges mentioned targeted support for women’s leadership, for example in the form of a direct invitation to apply for a job opening, as a relatively simple measure that could increase women’s representation in higher positions:

Job advertisements simply must be written in such a way that it is clear that women are welcome. You have to address them directly. […] The system, in order to be egalitarian, must treat women a little differently than men. And this is absolutely unacceptable to them [the men]. So I think that [it is needed to] actively seek women and actively address them.

An apex court judge; middle-age generation

To summarise, the research participants mostly blame family obligations and the lack of ambition on part of women as the two key factors for the vertical gender segregation of the Czech judiciary. The barriers that could be potentially remedied within the judiciary, like the nontransparent policy for promotion to higher courts (and its consequences) or the lack of targeted support for women’s leadership, were mentioned less often. That means that female judges tend to see the crucial reasons why women are underrepresented in the top echelons of the judiciary as being mostly outside of the judicial system itself.

### Roots of the Barriers and Obstacles: Nature, Choice, or Oppression?

Besides the perceived barriers to women’s promotion in the Czech judiciary, we were also interested in how the interviewed judges interpret their roots and causes. In this case we did not ask direct questions, but rather analysed the discourse they used to describe the key obstacles: the family obligations and the (expected) role of women as caregivers and household managers, and the lack of ambition.

#### Views on Gendered Division of Labour

When it comes to the interpretation of gender norms governing family obligations and paid and unpaid work, three positions could be identified. Some judges see the norms determining that women are primarily responsible for raising the children and running the household as natural; for others, it is a matter of their free choice; and yet others interpret these norms as oppressive and discriminatory. In general, however, the majority of the interviewed judges interpreted the status quo more or less as a fact, without questioning it, considering whether this arrangement is fair, or envisioning a different setup.

First, the statements of roughly a quarter of the participants indicate that they see the prevailing gender norms as *natural* and even desirable. They hold an essentialist view and see men and women as inherently different, with different traits and characteristics, and with different social roles and life missions: first and foremost, women are mothers and caregivers. These (traditional) attitudes were more prevalent among judges in higher age groups and from the top echelons of the judiciary:

After all, a woman is biologically adapted to motherhood and family care. I know it’s now levelling off, but I still think that the natural state of affairs is as nature made it.

An apex court judge; higher-age generation

I don’t think a woman should sit in a cave today, process leather, and keep the fire going. But it has always been the case that man feeds the family, and woman somehow cements and cares for the family.

An apex court judge; higher-age generation

According to some of the interviewed judges, the underrepresentation of women in the top echelons of the Czech judiciary is legitimate, natural, and even beneficial, given the different capabilities of men and women. They see women as less ambitious, more emotional, and less able to handle pressure, which are not very suitable traits for judges in general and those in leading positions in particular:

I would like to see more men here [in the judiciary] though […] Women are far more emotional […] And that’s why I think that a man is capable of greater foresight than a woman. […] So the pressure is enormous, and when it comes to a prestigious or a closely followed court case and the press is chasing you, and really the pressure is extraordinarily great […] and women, maybe this is what they can’t handle, because they’re far more emotional, by nature, so they perhaps cannot handle it. And in this situation, forcing someone to be here who doesn’t have what it takes...

An apex court judge; higher-age generation

I feel that women are women and men are men and that there are some reasons for that and that those natures are just different. And that it is not a social construct. And that to be in a leading position, you have to have balls, in the sense that you mustn’t mind criticizing the work of others […]. And I don’t think a lot of women have that ambition.

A district/regional/municipal court judge; younger generation

The interpretation of vertical gender segregation as a natural state of affairs precludes admitting that it could be a consequence of inequality, stereotypes, prejudice, or even discrimination. Some of the interviewed judges explicitly rejected the possibility that the lower representation of women in positions of power could be the consequence of gender-specific barriers and obstacles, despite the fact that they acknowledged that women are in a more difficult position due to childcare and household duties. Paradoxically, they pointed out that nothing prevents female judges from obtaining career advancement if they have such an ambition, and denied that there could be any discriminatory mechanisms at play:

After all, I still think that a man and a woman are historically, biologically rooted somewhere, and when women don’t want to go there [to higher positions] themselves, and I don’t think anyone here in this republic is preventing them from doing it. […] if a woman is capable enough, if she wants, she gets anywhere she wants, just like a man […]—so the voices that women don’t do it [strive for a promotion] because they would be discriminated against, that sounds silly to me.

An apex court judge; higher-age generation

I don’t like the talks that women are prevented from [career advancement], that they don’t have the opportunities, they don’t have the options, I don’t like to hear that, because I don’t think so […] I don’t think that if a woman wants to do something, that she can’t and that anything is stopping her.

An apex court judge; middle-age generation

The second group of participants shared and internalised the dominant gender norms; still, they emphasised that it is their personal preference, and other women’s preferences may be different. The accent on *free choice* sets them apart from the previously mentioned groups of judges who see the roles of men and women as naturally and inherently different:

Of course, each family has a different model, and our model, even if I praise my husband, but it is still the case that, even if we try to share it evenly, that I feel that I want to bear, I probably want to bear, more responsibility, that I have the feeling that I need to organise it [the family and the household] more. But that’s my personal setting; there may be women who have a different setting.

A district/regional/municipal court judge; younger generation

The third group of participants interpreted the dominant gender norms as *oppressive*. Interestingly, despite the clear-cut picture of gender inequality in the Czech Republic, they were in the minority: only four out of 14 judges reflected on broader questions of equality and fairness at the societal level at some point in the interview. They acknowledged the structurally unequal position of men and women in Czech society, particularly gender norms, stereotypes, and discrimination, as the root cause of vertical gender segregation in the Czech judiciary:

[…] women are in society, in the Czech Republic certainly, automatically discriminated against […].

An apex court judge; middle-age generation

I think the first reason is motherhood. In the beginning, we all have the same position and, maybe it’s because here in the Czech Republic, here we usually spend at least 2 years on maternity or parental leave, and the societal pressure, at least what I experienced when my female friends placed their children at the age of two in kindergartens, they were taken for “krkavčí matky” (raven mothers),^11^ and there was enormous pressure on them that the children would be stupid and socially destroyed and so on.

A district/regional/municipal court judge; younger generation

I still feel that our society is very much based on stereotypes. It is believed that women should be more inclined to take care of children, and of cooking and the household, and to pass it all on to the help of housekeepers and cooks is not yet habitual in this society. There are certainly women who do this; the thing is that they are not always looked at well.

An apex court judge; higher-age generation

#### Women’s Fault: Lack of Ambitions

Besides the perception of gender norms governing the division between paid and unpaid work in the household, we also analysed how the judges interpreted the second crucial obstacle to promotion they identified: lack of ambition. Again, the interviews revealed that most participants accept the *status quo* as granted and see the roots for the low representation of women in positions of power to be, in part, on the women themselves. Several judges claimed that women are not very interested in a career or that they do not have ambitions, without further consideration of deeper reasons:

I don’t know, I don’t think women are very interested in it [the position of president of the court].

An apex court judge; higher-age generation

[I]n my social circle, where I have friends, university graduates […] so those girls don’t have ambitions, they just don’t. And they’re just as smart, if not smarter than boys, just as capable.

A district/regional/municipal court judge; younger generation

A minority of interviewed judges, on the other hand, pointed out that overly ambitious and self-asserting women are frowned upon in Czech society. Judges in general are expected to “wait humbly” to be noticed and promoted, and while this seems to be a pattern that applies to men as well, we argue that due to gender norms social pressure to comply with this expectation is stronger in the case of female judges. Women are expected to not show too much ambition, otherwise, they are labelled as “careerists”, as explained by some of the research participants:

And it still seems to me that it is not very common for women to strive for a higher position. What I have experienced is that it is assumed that such a woman is a careerist and that she is weird. In the case of men, it is somehow taken more naturally, career advancement, the expression of one’s own opinion, to say to myself, I am good and I want to move on in my career.

A district/regional/municipal court judge; younger generation

So I will tell it how it is, it is taken so that when a person asks too much, it is considered as trying too hard to get the place [at a higher court] which is not appropriate, one should rather humbly wait, so I humbly wait.

A district/regional/municipal court judge; younger generation

The culture of waiting patiently that prevails in the Czech judiciary is also evident from how the female judges described their own career paths and plans. The judges that were not promoted throughout their career explained it by “not getting an offer”, and those who hold a position at a higher court than a district court mentioned that they “were offered” or “were asked” to advance to a higher court. One of the judges mentioned that she was approached with a promotion offer in a situation when all the male adepts had rejected it, and there was no one to fill the position. Several others interpreted their promotion not as a sign of their abilities but as a stroke of luck or a consequence of being in the right place at the right time. This, too, adds to the picture of how female judges, and women in general, perceive and present their career success.

Interestingly, some of the judges implicitly presented it as a virtue that they were approached with an offer and were not actively trying to advance their careers. Thus, they see the obstacles in promotion as being in women themselves, claiming that they are not ambitious enough, and still, they present the lack of ambition or at least their effective hiding of it as a value:

So I didn’t have to force the issue, no concessions, as I would say, my whole life’s work has paid off in the fact that it wasn’t me who had to apply, but […] I was asked.

An apex court judge; higher-age generation

At the same time, it must be stressed that showing ambition is indispensable in the judiciary. In the case of promotion to managerial posts, women have to find the courage to apply for them in open competition (Derka 2017), and in case of promotion to a higher court, they need to stand out to get noticed and be given an offer. Thus, female judges are trapped in a vicious cycle: being overly ambitious is seen as improper; they must not act like they are trying too hard, and they are expected to wait humbly. However, if they comply with the unwritten requirement of patient waiting, it seems that they lack ambition and, consequently, that the underrepresentation of women in positions of power is caused by their own lack of interest.

## Conclusion

The interviews with Czech female judges suggest that the deep underlying causes of vertical gender segregation in the Czech judiciary lie at the broader societal level, in the gender norms that translate into the division between paid and unpaid labour and societal expectation of who, when, and how should show ambition. In addition, several specific features of the Czech judicial system, most notably the nontransparent promotion policy and the lack of targeted support for women’s leadership, represent additional barriers to the career progression of female judges.

In general, judges were able to describe in detail various reasons for the underrepresentation of women in leadership positions. These reasons are consistent with the rich literature from around the world; so, the barriers themselves do not seem to be specific to the Czech Republic or to the CEE region. In line with previous research (e.g., Bonelli 2015; Boigeol 2013; Bonelli 2015; Chan 2014; Durant 2004; Duarte et al. 2014; Halilović and Huhtanen 2014; IDLO 2018; Schultz 2013; Schultz 2003; and references therein; Achmad and Halimatusa’diyah 2022; Kalem 2020; Shen 2020), mothers and primary carers (who are overwhelmingly women) face significant challenges having to do with work-life balance. The usual intense involvement in domestic labour means that a significant proportion of female judges may be so busy that they are happy when they can manage the job they already have. It is also more difficult for women to move to another city to get a more prestigious job.

Furthermore, in line with existing scholarship (Chan 2014; Kalem 2020; Shen 2020; Zheng et al. 2017), women have less time to do extra work that would make them stand out from the crowd and to build informal relationships. Both of these are important, especially when there are no formal rules (in the Czech case, this concerns advancement to higher courts). This supports the findings of previous studies pointing out that the lack of transparency puts women at a disadvantage (Shaw 2008; Valdini and Shortell 2014; Schultz and Virtue Foundation cited in IDLO 2018).

In addition, open ambition in a woman is still, to an extent, frowned upon in Czech society, including the judiciary. It could be interpreted as a sign of “trying too hard” and this could backfire on them as, according to the role congruity theory, deviating from expected gender roles (e.g., of being restrained, friendly, modest, and empathetic) increases the risk that women experience negative reactions and noncompliance (Eagly and Karau 2002). As a result, women are trapped: they have to show ambition if they want to get promoted and hide it at the same time.

While previous studies have noted “inner career obstacles and career renunciation” (Schultz 2013, 161–63) or “a genuine lack of interest in reaching a top position” (Boigeol 2013, 131) among women judges, their focus has not been on the question to what extent women judges perceive this as a gender-based disadvantage. This paper has probed this issue. Although the research participants were able to describe in detail the barriers faced by women judges, they are divided—in three ways—as to their interpretation. Majority of them do not reflect on the underlying causes and do not question the *status quo*. They mostly perceive the gender norms that designate women as being primarily responsible for family and household as natural, and, in consequence, they interpret the career impact of these norms as an inevitable part of a woman’s destiny. Alternatively, they see the adoption of these norms as a matter of their free choice, and reproduce the “choice” myth, according to which women have to choose between work and family (Smith 2015). Naturally, they then perceive the problems that stem from alignment with these gender norms as their own failures and inadequacy caused by their own behaviour and choices. Thus, these two positions lead to rather uncritical acceptance and lack of demand for a structural remedy. Only the third group of judges perceived the identified gender barriers and obstacles as unfair, hinting at the need to address them institutionally. At the same time, we did not identify any characteristics that these women have in common that would explain their feminist attitudes: they differ in age, position in the judicial hierarchy, place of study (in the Czech Republic or abroad), and whether or not they have children.

At large, the diversity of opinions on gender issues and their representation among women judges generally reflects the views present in Czech society. To this day, Czech society still holds rather traditional beliefs regarding male and female roles, according to which women are predominantly (natural) caregivers and men (natural) breadwinners (CVVM 2020). Also, when the Czech MPs—directly elected representatives of society—were asked whether they considered themselves feminists, only 50 out of 200 answered the poll question (Ježková 2022). The main part of the MPs, and also the main part of women MPs, answered that they did not consider themselves feminists (44%), a third answered evasively, and only a fifth answered positively. Thus, the reluctance to openly espouse feminist ideas is widespread in the Czech Republic, and women judges are definitely not on the front lines when it comes to feminism.

However, Czech women judges are not unique in their prevailing gender blindness. The lack of feminist consciousness and the resenting of feminism seem to be typical features of the whole CEE region (Dawn Metcalfe and Afanassieva 2005; Jezerska 2003). It should also be added that the reluctance to embrace feminist identities was also noted by the scholars examining the attitudes and perceptions of women judges in Australia (McLoughlin 2021), Portugal (Duarte et al 2014), China (Shen 2020), Brazil (Bonelli 2015) and Venezuela (Roche 2003).

How can we explain such resignation to ‘women’s fate’ and often quite essentialist and fatalistic views, especially in the case of a group of highly educated professionals? First, these attitudes can be a part of the historical legacy of the emancipation project under state socialism. This framed women’s equality through their equal role in economic production, not through their individual rights (Pascall and Kwak 2010). Since women’s participation in the judiciary is not itself a problem in the Czech Republic (their representation reaches 61%; Havelková et al. 2022), some women judges (especially those in the higher age group) may feel that the emancipatory mission—according to a definition valid for several decades—has been accomplished. This may be the reason why, although they were able to name the barriers, they perceived them as natural and not discriminatory.

Second, questioning, challenging, and resisting gender norms and an existing system takes time and energy which women—already burdened by the double shift of housework and paid work—often lack. According to the distinction suggested by Molyneux (1985), they focus on their practical gender interests (immediate perceived need) rather than their strategic ones that aim at changing the social order (for Czechia, see Maříková 2008).

Third, the acceptance of the status quo may have something to do with the conservative nature of the judicial profession (Feenan 2008). Judges are trained to protect the existing order and prevailing norms, and to apply the law without much asking and challenging. Contesting the deeply entrenched gender norms that underpin the social order may therefore be more troubling for them than for some other professions.

In consequence, we argue that the rationalisation of the underrepresentation of female judges in the top echelons of the Czech judiciary, the lack of reflection on its deeper reasons, and, in some cases, even the denial of the existence of any gender inequality contribute to the status quo and help to reproduce it. These findings also support previous studies that have concluded that “women are not a proxy for feminism” (Hunter et al. 2015, 579), and, consequently, simply adding more women to the judiciary is not a universal cure for a perceived lack of diversity (Bartlett and Douglas 2018).

A certain limitation of this study is that, like most studies on gender barriers in the judiciary, it is based only on interviews with female judges. Although it is undoubtedly critical to explore the perspectives of those who have first-hand experience with the gender barriers, interviewing men is another important research venue. This is not because men judges can be expected to have a deeper insight into gender relations in the judiciary compared to women. But, as they are the ones who hold the power in the judiciary and benefit from the *status quo*, a closer exploration of their perspectives on gender relations and their gender awareness may be useful in future considerations on how to bring about change and remove the gender barriers that Czech women judges currently face. After all, to make a feminist difference, feminist judges rather than women judges are what might be needed (McLoughlin 2021).

## Footnotes

1 Ireland 39%, Northern Ireland 36%, England and Wales 25%, Scotland 26% (CEPEJ 2022); Canada 44% (Statistics Canada 2022), Australia 41% (AIJA 2021), New Zealand 40% (The Office of the Chief Justice of New Zealand 2022), USA 34% (NAWJ 2022).

2 The International Development Law Organization recently summarised these as having to do with (a) selection and appointment processes; (b) challenges with work-life balance; (c) denial of opportunities that contribute to development and promotion (IDLO 2018).

3 Article 71(3) of Law No. 6/2002 Coll., on Courts and Judges.

4 Article 71(2) of Law No. 6/2002 Coll., on Courts and Judges.

5 Article 122 of Law No. 150/2002 Coll., the Code of Administrative Justice.

6 Article 71(2)-(3) of Law No. 6/2002 Coll., on Courts and Judges; and Article 122 of Law No. 150/2002 Coll., the Code of Administrative Justice.

7 Article 73(3) of Law No. 6/2002 Coll., on Courts and Judges; and Article 124 of Law No. 150/2002 Coll., the Code of Administrative Justice.

8 Instruction of the Ministry of Justice of 22 December 2010, No. 215/2010-OJ-ORGV, on the Nomination and Appointment of Court Officials.

9 Instruction of the Ministry of Justice of 25 July 2014, No. 100/2014–OJ-ORGV/3, on the Nomination and Appointment of Court Officials.

10 The study received ethical approval from the Central University Research Ethics Committee of the University of Oxford (Reference no. R63307/RE001) and the Masaryk University’s Ethics Committee (Reference no. EKV-2015–01).

11 This derogatory term is used in Czech to describe “bad” mothers who “abandon” their children and place them in day care in pursuit of careers.
